# The Association of High-Frequency Nut Intake With a Low Risk of Psychological Problems in Female Methamphetamine Users

**DOI:** 10.3389/fpsyt.2022.878859

**Published:** 2022-08-15

**Authors:** Zihong Song, Fang Dong, Yizhi Liu, Guanhua Liu, Baohua Li, Xiuyu Pang, Kang An, Dong Li, Shanshan Chen, Weijia Xing, Xizhu Xu

**Affiliations:** ^1^School of Public Health, Shandong First Medical University and Shandong Academy of Medical Sciences, Tai’an, China; ^2^The Second Affiliated Hospital of Shandong First Medical University, Tai’an, China

**Keywords:** methamphetamine (MA), female, psychological problems, diet, China

## Abstract

**Background:**

Recent years have witnessed a gradual increase in the number of female methamphetamine users. Meanwhile, female methamphetamine users are more likely to have psychological problems than male methamphetamine users. The association between diet and psychological problems have been found among non-methamphetamine user. The present study aims to investigate the relationship between dietary intake frequency and psychological problems in female methamphetamine users.

**Materials and Methods:**

A total of 109 female methamphetamine users, collected from a Compulsory Isolated Drug Rehabilitation Centre in northern China, participated in the study. All participants completed the Symptom Checklist 90 (SCL-90) questionnaire to assess psychological status. The relation of dietary intake frequency with the SCL-90 score was tested in partial correlation analysis. Multivariable regression models were used to calculate odds ratios to evaluate the association of dietary intake frequency with psychological problems.

**Results:**

Of the current female methamphetamine population, 33 participants were diagnosed with psychological problems using SCL-90. In the terms of dietary intake frequency, the frequency of nut intake in the psychiatric symptom group was significantly lower than that in the asymptomatic group. However, there was no difference in the frequency of other food intakes between the two groups. The frequency of nut intake was negatively correlated with the total score of SCL-90 and 8 different symptom clusters of psychopathologies on SCL-90. Logistic regression analysis indicated that the increased frequency of nut intake was associated with a lower risk of psychological problems.

**Conclusion:**

In the female methamphetamine population, increasing the frequency of nut intake may reduce the risk of psychological problems for female methamphetamine users.

## Introduction

Methamphetamine (MA), a new drug commonly known as “ice,” affects the activities of the peripheral nervous system and central nervous system (1). MA can lead to strong psychological dependence, neurotoxicity, and a high relapse rate and long-term abuse of MA can seriously damage the physical and psychological health of people (2). According to the official report, there were approximately 269 million drug abusers in the world in 2018, an increase of 30% over 2009; besides, vulnerable and marginalized groups, such as young people, women, and the poor, paid a price for the global drug problem (3). By the end of 2019, there had been 2.148 million drug users in China, accounting for 0.16% of the total population; MA is still the most abused drug in China (4). Traditional intravenous heroin has been unable to meet people’s needs, and drugs that can snore, inhale, or swallow have become the first choice, amphetamine and methamphetamine abuse prevail, and 2C-T-7 [2,5-dimethoxy-4-(n)-propylthiophenethylamine] has also emerged in networks with streets, with the use of networks for the evaluation of drug abuse markets to provide easily accessible and reliable information for professionals in the field of drug addiction (5, 6). In addition to “traditional” psychoactive substances, new psychoactive substances (NPS) have attracted increasing international attention, exhibiting neuropsychiatric and cardiovascular toxicities with potentially serious psychiatric and physical consequences that can lead to death (7).

Substance-induced psychosis (SIP) occurs in patients with the first episode of psychosis. Patients with SIP are at risk of developing a severe psychiatric disorder, schizophrenia spectrum disorder, or bipolar disorder, and the first attempt at a diagnosis of psychotic symptoms by Giovanni Martinotti (8) proposed substance-related exogenous psychosis may have important implications for the choice of therapeutic interventions. In postmodern adolescents, this is often accompanied by an irritable mood. In this case, the psychoactive substance acted as a powerful catalyst, enhancing this transient experience of excitement. Previous studies have shown that MA abusers often have negative emotions such as long-term anxiety and depression during abstinence (9–11). These psychological problems may aggravate the craving for MA, increase the addiction to MA (12) and increase the risk of relapse (13). There are also individuals who use MA have aggressive and violent tendencies (14). The psychological problems of MA users may directly lead to violent crimes, and thus increase the incidence and mortality risk of MA users (15). All of these negative emotions would weaken the motivation for rehabilitation therapy (16).

Female MA users are a vulnerable group as they are highly compliant and more vulnerable to the external environment (17). Previous research indicated that female MA users were more likely to show pleasant feelings after using MA than male MA users (18), which can also account for their higher MA addiction than male MA users (19). During the withdrawal period, female MA users have more severe withdrawal symptoms than male MA users (20), which greatly increases the possibility of relapse (21).

Historically, nutrition has been considered to be a factor leading to poor psychological health, considering that the central nervous system needs key nutrients to maintain optimal function. Several research demonstrated that there is a close relationship between nutrition and the psychological health of adults (22–26). Therefore, we conceived a hypothesis: the diet of MA users might have a close correlation to their psychological health.

As far as we know, there has been no study to explore whether or not the psychological problems among MA users are related to the frequency of dietary intake. As we all know, MA users are often accompanied by a high prevalence of psychological problems. Previous analysis of the causes of the high prevalence of psychological problems found that MA-induced psychosis was caused by cortical injury, which was caused by the increased release of dopamine (DA) in the striatum over time (27). It was suggested that the psychiatric symptoms of amphetamine-type stimulants may be related to high frequency (28) and high dose (29). At the same time, a report on the relationship between the starting age of amphetamine use and the risk of psychiatric hospitalization shows that the starting age of stimulant use affects the occurrence of psychiatric symptoms (30). However, there has been no study to explore whether the psychological problems among female MA users are related to dietary intake frequency. A 2-year clinical follow-up was conducted to evaluate the role of a Mediterranean diet supplemented with extra virgin olive oil or nuts in preventing the risk of recurrence of monophasic depression (31). A survey of a young adult Appalachian college population found that improving college students’ dietary intake by increasing access to healthy food can improve students’ psychological problems (32). In addition, it has been found that the dietary intake of males and females with psychological health disorders is different, and females with depression or anxiety consume more unhealthy food than males (22–24). However, whether the psychological problems among female MA users are related to dietary intake frequency remains unknown. Therefore, the purpose of this study is to explore the relationship between psychological problems and dietary intake frequency among female MA users.

In this study, we would clarify whether there is a close relationship between dietary intake frequency and psychological problems among female MA users in the hope of providing a scientific theoretical basis for reducing and alleviating the psychological problems of female MA users.

## Materials and Methods

### Participants

A total of 109 female MA users were recruited from a Compulsory Isolated Drug Rehabilitation Centre in northern China. All female MA users were screened using MA dependence criteria from the Diagnostic and Statistical Manual of Mental Disorders (DSM-5). SCL-90 total score ≥ 160 refers to the psychotic symptoms group, SCL-90 total score <160 refers to the asymptomatic group. All participants participated voluntarily and signed informed consent.

The inclusion criteria are (a) female; (b) age ≥ 18 years; and (c) MA users. The exclusion criteria are (a) mental or neurological diseases (such as schizophrenia, affective disorders, stroke, epilepsy, or Parkinson’s syndrome); (b) patients who have been diagnosed with major physical diseases such as cerebrovascular, cardiovascular, or neurodegenerative diseases.

The demographic information of each participant was obtained through one-on-one questioning by the investigators, who went through standardized training. In this study, all private information was encoded and restricted.

### Demographic/Methamphetamine Abuse History/Dietary Intake Frequency Form

The questionnaire includes demographic characteristics, MA abuse history, and dietary intake frequency. Demographic characteristics include age, marital status, education level, etc. MA abuse history includes MA dose (g), admission time (month), duration of methamphetamine users (years) and frequency of drug use. Dietary intake frequency includes the intake frequencies of milk, beans, vegetables, fruits, nuts, seafood, eggs, meat, and sugary drinks.

### Symptom Checklist-90

Symptom Checklist-90 (SCL-90) which showed favorable efficiency in previous studies was used in this study with the total score indicating the overall mental health of drug users (33, 34). It has 90 items, accessing 9 different symptom clusters of psychopathologies, including somatization (SOM); obsessive–compulsive behavior (O-C); interpersonal sensitivity (I-S); depression (DEP); anxiety (ANX); hostility (HOS); phobic anxiety (PHOB); paranoid ideation (PAR); and psychoticism (PSY). Each of the 9 different symptom clusters of psychopathologies is assessed with 6–13 items. SCL-90 total score ≥ 160 was suggestive of potential psychological health issues.

### Statistical Analysis

All data were analyzed using SPSS 22.0. Categorical variables are reported as a proportion (%). Group differences were compared using the Student’s *t*-test for continuous variables and χ^2^ test for categorical variables. A partial correlation analysis was used to evaluate the correlation between the dietary intake frequency and the total and index scores of SCL-90 in the MA population. Multivariable regression models were used to analyze the influence of dietary intake frequency on psychological problems of female MA users with SCL-90 score as the dependent variable. All tests were performed by two-sided tests, with *P* < 0.05 indicating statistically significant differences.

## Results

### Demographic Characteristics, Methamphetamine Abuse History, and Dietary Intake Frequency of the Participants

As shown in Table 1, there are no differences in age, marital status, methamphetamine dose, admission time, duration of methamphetamine users, and other variables between the two groups, but there are differences in frequency of drug use. The difference analysis of dietary intake frequency between the two groups showed that there was a difference only in the frequency of nut intake between the two groups, and the frequency of nut intake of the psychotic symptoms group was significantly lower than that of the asymptomatic group. However, there was no significant difference in the intake frequency of other food types between the two groups.

**TABLE 1:** Demographic characteristics, MA abuse history, and dietary intake frequency of the participants.

Variable	Total (n=109)	Asymptomatic group (n=76)	Psychotic symptoms group (n=33)	*P*
Age (year)	30.08 ± 5.30	29.99 ± 5.03	30.30 ± 5.94	0.776
Urban, n (%)	94 (88.68)	68 (88.31)	26 (89.66)	0.846
Unemployed, n (%)	31 (29.25)	22 (28.57)	9 (31.03)	0.804
Income<3000yuan, n (%)	32 (29.36)	21 (27.63)	11 (33.33)	0.548
Single, n (%)	96 (88.07)	66 (86.84)	30 (90.91)	0.547
Alcohol drinking, n (%)	71 (65.14)	47 (61.84)	24 (72.73)	0.273
Cigarette smoking, n (%)	89 (81.65)	62 (81.58)	27 (81.82)	0.976
NO-exercise, n (%)	58 (53.21)	39 (51.32)	19 (57.58)	0.547
Methamphetamine dose (g)	0.46 ± 0.57	0.50 ± 0.66	0.37 ± 0.27	0.267
Duration of methamphetamine users (year)	4.39 ± 2.32	4.58 ± 2.41	3.94 ± 2.08	0.188
Admission time (month)	7.69 ± 2.87	7.64 ± 2.93	7.79 ± 2.78	0.812
Frequency of drug use, n (%)				0.042
1 time/week	27 (24.77)	21 (27.63)	6 (18.18)	
2-5 times/week	41 (37.61)	32 (42.11)	8 (24.24)	
1-2 times/day	18 (16.51)	9 (11.84)	10 (30.31)	
More than 3 times/day	23 (21.10)	14 (18.42)	9 (27.27)	
**Dietary intake n (%)**				
Beans				0.801
<1 time/week	55 (50.46)	39 (51.32)	16 (48.48)	
1-7 times/week	35 (32.11)	23 (30.26)	12 (36.36)	
>7 times/week	19 (17.43)	14 (18.42)	5 (15.15)	
Vegetables				0.692
<1 time/week	14 (12.84)	11 (14.47)	3 (9.09)	
1-7 times/week	31 (28.44)	22 (28.95)	9 (27.27)	
>7 times/week	64 (58.72)	43 (56.58)	21 (63.64)	
Fruits				0.604
<1 time/week	9 (8.26)	5 (6.58)	4 (12.12)	
1-7 times/week	19 (17.43)	13 (17.11)	6 (18.18)	
>7 times/week	81 (74.31)	58 (76.32)	23 (69.70)	
Milk				0.563
<1 time/week	33 (30.28)	25 (32.89)	8 (24.24)	
1-7 times/week	30 (27.52)	19 (25.00)	11 (33.33)	
>7 times/week	46 (42.20)	32 (42.11)	14 (42.42)	
Nuts				0.028
<1 time/week	41 (37.61)	23 (30.26)	18 (54.55)	
1-7 times/week	50 (45.87)	37 (48.68)	13 (39.39)	
>7 times/week	18 (16.51)	16 (21.05)	2 (6.06)	
Seafood				0.284
<1 time/week	46 (42.20)	33 (43.42)	13 (39.39)	
1-7 times/week	34 (31.19)	26 (34.21)	8 (24.24)	
>7 times/week	29 (26.61)	17 (22.37)	12 (36.36)	
Eggs				0.749
<1 time/week	24 (22.02)	18 (23.68)	6 (18.18)	
1-7 times/week	31 (28.44)	22 (28.95)	9 (27.27)	
>7 times/week	54 (49.54)	36 (47.37)	18 (54.55)	
Meat				0.752
<1 time/week	22 (20.18)	16 (21.05)	6 (18.18)	
1-7 times/week	31 (28.44)	20 (26.32)	11 (33.33)	
>7 times/week	56 (51.38)	40 (52.63)	16 (48.48)	
Sugary drinks				0.482
<1 time/week	18 (16.51)	11 (14.47)	7 (21.21)	
1-7 times/week	13 (11.93)	8 (10.53)	5 (15.15)	
>7 times/week	78 (71.56)	57 (75.00)	21 (63.64)	

*Continuous and categorical variables were expressed as the mean _ SD and percentage (%). Student’s t-test and $2 test were used to probe for differences in continuous variables and categorical variables. In the MA population, the total score of SCL-90 _ 160 for was the psychotic symptoms group, and the total score of SCL-90 < 160 was for the asymptomatic group.*

### Correlation Between Symptom Checklist-90 Score and Dietary Intake Frequency

**FIGURE 1:**
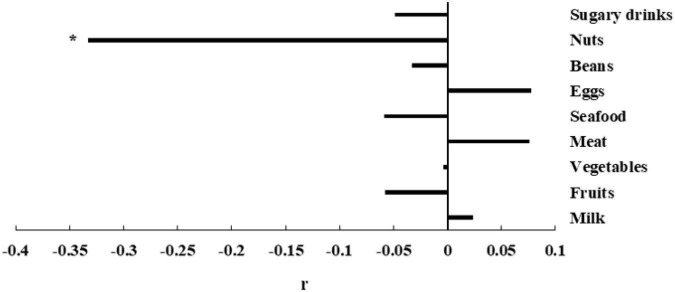
The association between SCL-90 and dietary intake frequency. Partial r and *P*-value were obtained after adjustment for the frequency of drug use. *P-value < 0.05.

In the partial correlation analysis model, the frequency of drug use was corrected. After correcting for the confounding factors, the correlation results between the two are shown in Figure 1. There is a negative correlation between the intake frequency of nuts and the total score (*r* = -0.333, *P* < 0.001). The correlation between the intake frequency of milk (*r* = 0.024, *P* = 0.806), fruits (*r* = − 0.058, *P* = 0.549), vegetables (*r* = −0.004, *P* = 0.968), meat (*r* = 0.076, *P* = 0.433), seafood (*r* = −0.059, *P* = 0.541), eggs (*r* = 0.078, *P* = 0.419), beans (*r* = −0.033, *P* = 0.734), and sugary drinks (*r* = −0.049, *P* = 0.613) and the total score of SCL-90 is not statistically significant.

### Correlation Between Symptom Checklist-90 Score and Nut Intake Frequency

**FIGURE 2:**
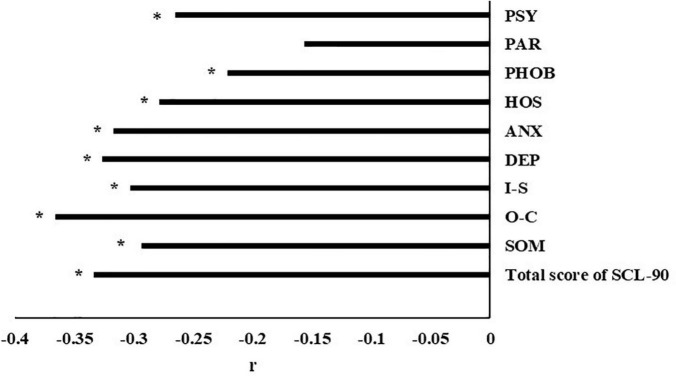
Correlation between total score of SCL-90 and different nut intake groups. Partial r and *P*-value were obtained after adjustment for the frequency of drug use. SOM, somatization; O-C, obsessive–compulsive behavior; I-S, interpersonal sensitivity; DEP, depression; ANX, anxiety; HOS, hostility; PHOB, phobic anxiety; PAR, paranoid ideation; PSY, psychoticism. *P-value < 0.05.

In the partial correlation analysis model, we corrected the frequency of drug use. After correcting for the confounding factors, the correlation results between the two are shown in Figure 2. the correlation between nut intake frequency and total score of SCL-90 (*r* = −0.333, *P* < 0.001), SOM (*r* = −0.293, *P* = 0.002), O-C (*r* = −0.366, *P* < 0.001), I-S (*r* = −0.303, *P* = 0.001), DEP (*r* = −0.326, *P* = 0.001), ANX (*r* = −0.317, *P* = 0.001), HOS (*r* = −0.278, *P* = 0.004), PHOB (*r* = −0.221, *P* = 0.022) and PSY (*r* = −0.265, *P* = 0.006) is negatively correlated, with statistical significance, and the correlation with PAR (*r* = −0.156, *P* = 0.107) is not statistically significant.

### Differences in the Scores of Symptom Checklist-90 and the Prevalence Rate of Psychological Problems Among Different Nut Intake Frequencies Groups

**FIGURE 3:**
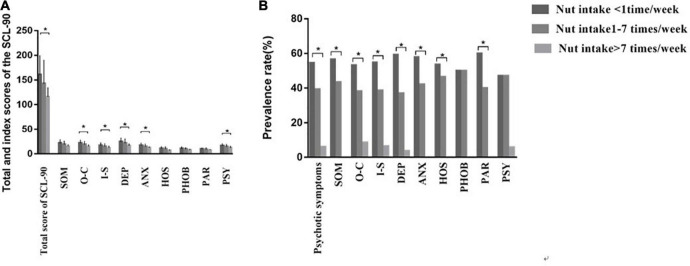
Differences of the SCL-90 among nut intake < 1 time/week, nut intake 1–7 times/week, and nut intake > 7 times/week. **(A)** Differences in total and nine different symptom clusters of psychopathologies scores of SCL-90 among different nut intake frequencies groups. **(B)** Differences in the prevalence rate of psychological problems among different nut intake frequencies groups. SOM, somatization; O–C, obsessive–compulsive behavior; I–S, interpersonal sensitivity; DEP, depression; ANX, anxiety; HOS, hostility; PHOB, phobic anxiety; PAR, paranoid ideation; PSY, psychoticism. *P-value < 0.05.

Comparing different nut intake frequency groups, SCL-90 scores included 9 different symptom clusters of psychopathology scores and a total score of SCL-90. Figure 3A shows that there are differences in total score, O-C score, I-S score, DEP score, ANX score, and PSY score among the three nut intake frequency groups, and the score decreases with the increase of nut intake frequency. There was no difference in the scores of SOM, HOS, PHOB, and PAR among the three groups.

The prevalence of psychological problems and nine different symptom clusters of psychopathologies among the three groups were analyzed. Figure 3B shows that there were differences in the prevalence of psychological problems, SOM, O-C, I-S, DEP, ANX, HOS and PAR among the three groups, and the prevalence increased with the decrease in nut intake frequency. There was no difference in the prevalence of PHOB and PSY among the three groups.

### Multivariable Logistic Regression Analysis of the Relationship Between the Frequency of Nut Intake and the Risk of Psychological Problems

**FIGURE 4:**
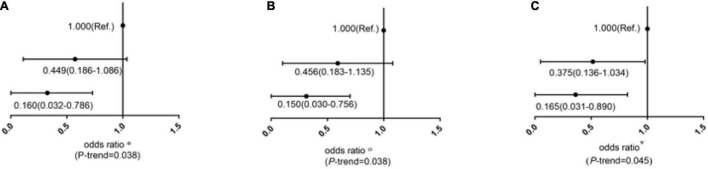
ORs (95% CI) of psychological problems according to the frequency of nut intake in cross-sectional study. **(A)** Was the factor without adjustment; **(B)** Multivariate ORs were adjusted for age, income, occupation, residence, and marriage; **(C)** Multivariate ORs were adjusted for age, income, occupation, residence, marriage methamphetamine dose, methamphetamine use, admission time, and frequency of drug use.

To explore the relationship between nut intake frequency and the risk of psychological problems, we established three models. First, in model 1, we did not make a factor correction. The results showed that in Figure 4A, the frequency of nut intake was < 1 time/week, which was used as the reference group. For the group with nut intake of 1–7 times/week, odds ratio (OR) (95% CI) was 0.449 (0.186–1.086), and for the group with nut intake > 7 times/week, OR (95% CI) was 0.160 (0.032–0.786).

In model 2, we corrected age, income, occupation, residence, and marriage. The results are shown in Figure 4B, the frequency of nut intake was < 1 time/week, which was used as the reference group. For the group with nut intake of 1–7 times/week, OR (95% CI) was 0.456 (0.183–1.135), and the for the group with nut intake > 7 times/week, OR (95% CI) was 0.150 (0.030–0.756).

In model 3, we corrected age, income, occupation, residence, and marriage, methamphetamine dose, methamphetamine use, admission time, and frequency of drug use. The results are shown in Figure 4C, the frequency of nut intake was < 1 time/week, which was used as the reference group. For the group with nut intake of 1–7 times/week, OR (95% CI) was 0.375 (0.136–1.034), and for the group with nut intake > 7 times/week, OR (95% CI) was 0.165 (0.031–0.890). That is, the risk of psychological problems decreased with the increase of nuts’ frequency.

## Discussion

In this study, we explored the correlation between psychological problems and dietary intake frequency of female MA users. The increased frequency of nut intake is associated with a reduced risk of psychological problems. The high frequency of nuts intake was the protective factor against psychological problems.

It has been revealed in our study that there are differences in the frequency of drug use between the asymptomatic group and the psychotic symptoms group. The rate of a high frequency of drug use in the psychotic symptom group is higher than that in the asymptomatic group. Similar to other literature reports (35), a high frequency of drug use is a risk factor for drug users suffering from depression. Meanwhile, Su et al. (36) reported that the frequency of drug use is a risk factor for anxiety in MA use during an acute withdrawal period.

Among the non-MA user population, studies have found that psychological problems are related to diet. For example, a large number of studies have considered the relationship between diet and human health, indicating that diet also has a potential effect on the risk of psychological problems (37, 38). A healthy diet (39) and Mediterranean diet (40) are associated with a lower risk of depression. Two recent clinical trials on psychological illness and psychological health (41, 42) show positive findings on the improvement and remission rate of depressive symptoms under the healthy diet program. In addition, in 2020, a literature review based on 37 studies confirmed the link between polyphenol intake and the risk of depression and the reduction of the severity of depressive symptoms (43). However, it is unknown whether the prevalence of psychological problems among MA users is related to diet.

It was found in our study that people with psychological problems had a lower frequency of nut intake than those without psychological problems. To some extent, this is consistent with previous studies by Su et al. (44) on Chinese adults depressed patients have lower nut intake. This study shows there was no difference in the intake frequency of milk, meat, beans, fruits, vegetables, seafood, eggs, and sugary drinks between the two groups. However, previous studies have shown people with depression or anxiety will exhibit lower fruit (45) intake and higher added sugar intake. Moreover, epidemiological studies have shown that beans can prevent depression (46, 47). In fact, there is empirical evidence that insufficient red meat intake is associated with a greater likelihood of depression or anxiety in females (48). This may be because most of the previous studies were conducted among non-MA user populations, with the females not being the only participants, but our study was conducted among female MA users, so there is no difference between the two groups.

This study found that there was a negative correlation between nut intake frequency and SCL-90 score, with the increase in nut intake frequency, the risk of psychological problems decreased, which suggests that we should increase the frequency of nut intake from the daily diet in female MA users. It was found that the frequency of nut intake was directly related to psychological problems, which was consistent with previous studies. For example, several studies in animals and humans have also shown that nuts in the diet have an impact on the structure, biochemistry, physiology, and function of the brain. Regular consumption of nuts is considered to play a healthy role in human brains, and cognitive and neuropsychiatric diseases (49–54). It is speculated that eating nuts may be beneficial for mental symptoms, mainly related to their various biological activities: such as the regulation of neurotransmitters (55, 56), anti-inflammatory effects (57), and changes in the central nervous system (49, 50, 54). Nuts are rich in n-3 polyunsaturated fatty acids, which have beneficial effects on health and can enhance neural plasticity and prevent neuron damage (58–62). Studies have found that n-3 polyunsaturated fatty acids can inhibit the inflammatory response of patients with depression, which indicates that nuts have a potential beneficial effect on depression (63–66). It has been found that long-term dependence on MA can lead to the injury of 5-hydroxytryptamine (5-HT) and DA nerve endings in the striatum, hippocampus, and prefrontal cortex, and even the apoptosis of 5-HT neurons and DA neurons (67, 68). However, animal model studies have shown that n-3 polyunsaturated fatty acids can reduce inflammation-induced neurogenesis to the same extent as antidepressants (69) and that n-3 polyunsaturated fatty acids can regulate neuroendocrine, serotonergic, and dopaminergic neuroendocrine transmission (70). In addition, nuts are rich in niacin, which may be a potential drug to support the treatment of schizophrenia (71). It is too early for niacin deficiency to be the cause of this progressive degeneration, but it seems that it may contribute to the neurodegenerative process because it can first cause neurodegeneration. It has been found that niacin particularly affects the posterior neural pathway of the cingulate cortex in schizophrenic patients (72). Most importantly, it is the precursor of nicotinamide adenine dinucleotides (NAD) and nicotinamide adenine dinucleotide phosphate (NADP). There is evidence that intracellular NAD plays a key role in metabolic regulation and repair, and the intracellular NAD concentration can be changed by nicotinic acid or nicotinamide administration (73). Nicotinamide has a neuroprotective effect at a pharmacological dose (74). This effect may be related to the pathogenesis or remission of schizophrenia. We can easily conclude that niacin is effective as an intensive treatment for patients with schizophrenia (75). Parletta’s et al. (41) dietary intervention in patients with depression found that the reduction of depression was associated with the increase of nuts. At the same time, a previous study showed that only the Mediterranean diet (nuts as a food group) was negatively correlated with depressive symptoms (76). Gignac’s et al. (77) research on pregnant women shows that nut intake in early pregnancy is associated with long-term children’s neuropsychological development.

Our study has important advantages. First of all, the participants in this survey did not receive drugs, psychotherapy, or other types of rehabilitation interventions in the Compulsory Isolated Drug Rehabilitation Centre in northern China, nor did they have variables that affected the results of the SCL-90 reported by the sample, nor did they affect the overall level of psychological problems. Secondly, this is a survey on the relationship between dietary intake frequency and psychological problems in the female MA population. This study shows that the high frequency of dietary intake, especially nuts, is a protective factor for psychological problems. Most importantly, these findings have laid a solid foundation for the investigation of dietary and psychological problems among female MA users. However, there are some limitations to be pointed out. Since all female MA users were recruited from a Compulsory Isolated Drug Rehabilitation Centre in northern China, the results only reflect the overall situation of female MA users in a Compulsory Isolated Drug Rehabilitation Centre in northern China. The investigation of diet in this study involves frequency only, and it can be combined with intake in future research.

In conclusion, in the study of the female MA population, there is a negative correlation between the frequency of nut intake and psychological problems. Increasing the frequency of nut intake can improve the psychological problems of female MA users, which gives us a very important hint: distributing nuts to female MA users every day in the Compulsory Isolated Drug Rehabilitation Centre and increasing their nut intake may reduce or alleviate their psychological problems. Nut cultivars are diverse, but they share many common nutritional characteristics. They are a good source of unsaturated fatty acids for daily life, in addition, they contain large amounts of plant sterols and polyphenolic compounds that are not found in animal foods (78, 79). Despite the great benefits of consuming nuts for psychiatric symptoms, whether any kind of nuts is more beneficial than others remains unknown. In this study, only the frequent intake of nuts was addressed, without indicating the specific kind of nuts. Further research is needed to determine how different kinds of nuts can help improve psychiatric symptoms.

## Data Availability Statement

The raw data supporting the conclusions of this article will be made available by the authors, without undue reservation.

## Ethics Statement

The studies involving human participants were reviewed and approved by Ethics Review Committee of Taishan Medical College. The patients/participants provided their written informed consent to participate in this study.

## Author Contributions

XX, FD, YL, GL, and ZS: study design. YL, BL, XP, DL, KA, SC, and ZS: data collection, analysis, and interpretation. ZS: manuscript writing. ZS, XP, XX, and WX: statistical analysis and administrative, technical, or material support, and supervision. XX and WX: critical revision of the manuscript for intellectual content. All authors approved the final version of the manuscript.

## Conflict of Interest

The authors declare that the research was conducted in the absence of any commercial or financial relationships that could be construed as a potential conflict of interest.

## Publisher’s Note

All claims expressed in this article are solely those of the authors and do not necessarily represent those of their affiliated organizations, or those of the publisher, the editors and the reviewers. Any product that may be evaluated in this article, or claim that may be made by its manufacturer, is not guaranteed or endorsed by the publisher.
